# An Early Cretaceous enantiornithine (Aves) preserving an unlaid egg and probable medullary bone

**DOI:** 10.1038/s41467-019-09259-x

**Published:** 2019-03-20

**Authors:** Alida M. Bailleul, Jingmai O’Connor, Shukang Zhang, Zhiheng Li, Qiang Wang, Matthew C. Lamanna, Xufeng Zhu, Zhonghe Zhou

**Affiliations:** 1 Key Laboratory of Vertebrate Evolution and Human Origins, Institute of Vertebrate Paleontology and Paleoanthropology, 142 Xizhimenwai dajie, 100044 Beijing, China; 2 CAS Center for Excellence in Life and Paleoenvironment, 100044 Beijing, China; 3 College of Earth and Planetary Sciences, University of Chinese Academy of Sciences, 100049 Beijing, China; 4 Section of Vertebrate Paleontology, Carnegie Museum of Natural History, Pittsburgh, PA 15213-4080 USA

## Abstract

Understanding non-crown dinosaur reproduction is hindered by a paucity of directly associated adults with reproductive traces. Here we describe a new enantiornithine, *Avimaia schweitzerae* gen. et sp. nov., from the Lower Cretaceous Xiagou Formation with an unlaid egg two-dimensionally preserved within the abdominothoracic cavity. Ground-sections reveal abnormal eggshell proportions, and multiple eggshell layers best interpreted as a multi-layered egg resulting from prolonged oviductal retention. Fragments of the shell membrane and cuticle are both preserved. SEM reveals that the cuticle consists of nanostructures resembling those found in neornithine eggs adapted for infection-prone environments, which are hypothesized to represent the ancestral avian condition. The femur preserves small amounts of probable medullary bone, a tissue found today only in reproductively active female birds. To our knowledge, no other occurrence of Mesozoic medullary bone is associated with indications of reproductive activity, such as a preserved egg, making our identification unique, and strongly supported.

## Abstract (web summary)

The fossil record of the reproductive traits of early birds is limited. Here, Bailleul and colleagues describe the Cretaceous enantiornithine bird *Avimaia schweitzerae*, which preserves an unlaid egg in the abdominal cavity and putative medullary bone.

## Introduction

In nearly every aspect, modern birds (neornithines) are highly modified from the typical reptilian condition^1^. Although many avian skeletal features were absent in stem birds (e.g., a keeled sternum, a beak, a pygostyle), new information is making it increasingly clear that many soft-tissue and behavioral features associated with extant birds had their origins among the Dinosauria^2^. Avian reproduction is no exception—birds are the only living amniotes with a single functional ovary and oviduct and are additionally characterized by proportionately large eggs^1^, a high degree of parental care^1^, and the use of medullary bone (MB), an ephemeral tissue that serves as a calcium reservoir for eggshell production during the reproductive cycle^3–5^. In the fossil record, adults directly associated with reproductive traces are extremely rare, but some key specimens have recently led to important discoveries about the evolution of the modern avian mode of reproduction. For example, preserved soft tissues interpreted as ovarian follicles suggest stem birds had already lost function of the right ovary^6,7^, and the histology of one specimen of *Tyrannosaurus rex* suggests that MB evolved well outside the avian clade^4,8^.

Here we describe an important new specimen of enantiornithine bird preserving a wealth of reproductive features and information, including a flattened intra-abdominal egg and traces of MB. The specimen comes from the Lower Cretaceous Xiagou Formation and represents a new taxon, *Avimaia schweitzerae* gen. et sp. nov. We investigate the preserved egg through standard paleohistological methods, scanning electron microscopy (SEM), and energy-dispersive x-ray spectroscopy (EDS). We discuss the implications of these findings in context of our understanding of dinosaur reproduction and the evolution of the specialized crown avian condition.

## Results

### Systematic paleontology

Aves Linnaeus, 1758

Pygostylia Chiappe, 2002

Ornithothoraces Chiappe, 1995

Enantiornithes Walker, 1981

*Avimaia schweitzerae* gen. et sp. nov.

### Etymology

The generic name *Avi-* (bird) *maia* (mother) refers to the fact the specimen is a female preserved with an egg in the body cavity*. Schweitzerae* is in honor of Mary Higby Schweitzer for her ground-breaking works on MB and for her role in establishing the field of molecular paleontology.

### Holotype

**Fig. 1:**
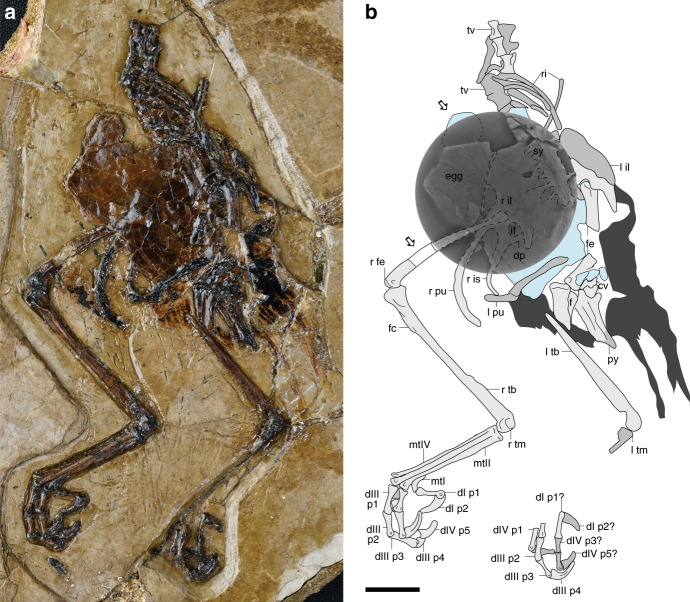
Photograph and line drawing of the holotype of *Avimaia schweitzerae*, IVPP V25371. **a** Photograph of the partial skeleton with feather impressions, and the crushed preserved egg between the pubes; **b** interpretive line drawing, with white arrows indicating the two fragments extracted for microscopic analysis with a super-imposed CT-scan revealing the egg and underlying elements of the right pelvis in dorsal (synsacrum) and medial (ilium) view. Gray denotes bones (darker gray indicating poor preservation), blue denotes the egg, and dark gray denotes feather impressions. cv caudal vertebra, d digit, dp dorsal process, f fibula, fc fibular crest, fe femur, if ilioischiadic foramen, il ilium, is ischium, l left, mt metatarsal, p pedal phalanx, pu pubis, py pygostyle, r right, ri rib, sy synsacrum, tb tibiotarsus, tm tarsometatarsus, tv thoracic vertebra. Scale bar is 1 cm

IVPP V25371 (Institute of Vertebrate Paleontology and Paleoanthropology), an articulated partial skeleton with some feather traces, consisting of the caudal half of the axial column, the pelvis, and the hind limbs, mostly exposed ventrolaterally (Fig. 1, Supplementary Fig. 1).

### Referred specimen

CAGS-IG-04-CM-007 (Institute of Geology, Chinese Academy of Geological Sciences), a semi-articulated partial skeleton consisting of pelvic fragments and nearly complete hind limbs^9^.

### Locality and horizon

Near Changma Village, Yumen City, Gansu Province, northwestern China; Lower Cretaceous (lower–middle Aptian) Xiagou Formation^10^.

### Diagnosis

Small-bodied enantiornithine (robust, cranially forked pygostyle, distal condyles of tibiotarsus contacting medially, J-shaped metatarsal I, metatarsal IV mediolaterally reduced relative to metatarsals III and IV, metatarsal IV trochlea reduced to single condyle; Supplementary Note 1) with the following autapomorphies: pubis delicate and strongly curved so that the caudal margin is concave throughout; distal end of ischium dorsally curved.

### Description

Seven thoracic vertebrae with spool-like centra precede the synsacrum, which consists of at least eight fully fused vertebrae (Fig. 1b). The synsacral neural spines, visible in the computed tomography (CT)-scans, form a low, continuous ridge on the dorsal surface (Fig. 1). Typical of enantiornithines, the pygostyle is craniodorsally forked proximally with ventrolateral processes extending most of its length^11^; the distal end is broken.

Fusion of the pelvis at the acetabulum cannot be determined. The cranial margin of the ilium is rounded and the dorsal margin is gently convex. The preacetabular process is longer and broader than the triangular postacetabular process (Fig. 1). Visible on the right, the dorsolateral surface forms a low ridge that is also present in *Sinornis*^12^ (Fig. 1b). The pubic peduncle of the ischium is broader than that of the ilium. The craniodorsally oriented dorsal process of the ischium contacts—but is not fused to—the ilium, demarcating an ilioischiadic foramen, as in *Qiliania* and PVL 4032–3 (Fundación-Instituto Miguel Lillo)^11,13^. The body of the ischium is distally tapered and curved dorsally. As in CAGS-IG-04-CM-007 ^9^, the pubic shaft is long and delicate and the distal third is strongly recurved such that it is orientated at nearly 90° from the main portion of the shaft (ventrally deflected in *Qiliania*^13^; robust and only weakly curved in *Feitianius*^14^). The distal ends are not fused but may have been in contact medially in life.

The femur is nearly straight with a posterior trochanter excavating its proximomedial surface, as in other enantiornithines^11^. The patellar groove is very weakly developed. The tibiotarsus is fully fused such that the ascending process of the astragalus cannot be discerned (forms a raised surface in *Qiliania*^13^). The proximocaudal surface bears two tubercles previously interpreted as the facies articularis lateralis and popliteal tubercle in pengornithids^14^. Proximomedial excavation is probably a diagenetic artifact. The fibular crest is short (14% the length of the tibiotarsus) and located well distal to the proximal articular surface. The distal condyles are large, round, and contact medially. The medial surface of the medial condyle is nearly flat (concave in *Qiliania*^13^). Only a fragment of the left fibula is preserved.

The distal tarsals are fully fused to the metatarsals, which are co-planar and unfused throughout their lengths. Metatarsal III is the longest; metatarsal IV is longer than II, which terminates level with the proximal end of the metatarsal III trochlea. Metatarsal II has a tubercle for the attachment of the m. tibialis cranialis located one-fifth from the proximal end on the craniolateral surface contacting metatarsal III. A deep intertrochlear incision is present between metatarsals II and III, probably resultant from post-mortem plantar displacement of metatarsal II. The metatarsal II trochlea is angled so that the medial margin is proximal to the lateral margin and the trochlear condyles are only weakly separated. The trochlea of metatarsal IV is reduced to a single condyle as in most enantiornithines^15^. The condyle is angled plantarolaterally, extending weakly onto the dorsal surface of metatarsal III just proximal to its trochlea. Metatarsal I articulates on the medial surface of metatarsal II as in all enantiornithines. Exposed in craniomedial view, it is J-shaped with the trochlear ramus measuring approximately two-thirds of the length of the metatarsal shaft, proportionally shorter than in other enantiornithines (half of the shaft length in *Qiliania*^13^, less in *Feitianius*^14^).

Pedal digit II is more robust than III and IV with a slightly larger ungual phalanx. The penultimate phalanx of digit II is 50% longer than the first phalanx. Digit III includes three long non-ungual phalanges, with the proximal and penultimate phalanges being subequal in length and longer than the intermediate phalanx. Digit IV is mostly not exposed.

Short body feathers are preserved in the caudodorsal region, extending over the pygostyle where they become slightly longer (Fig. 1). Elongate ornamental tail feathers appear to be absent.

### Egg microstructure

**Fig. 2:**
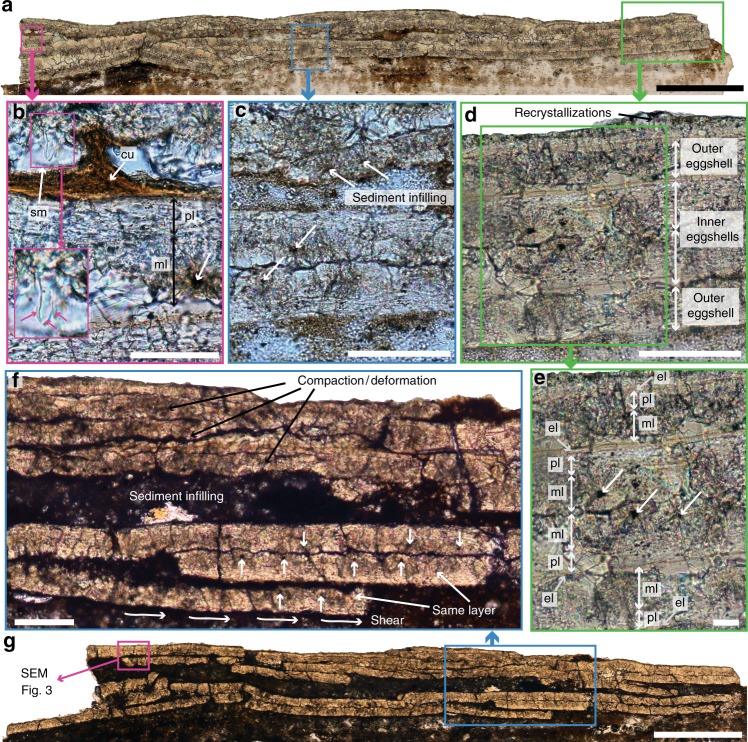
Light microscopy reveals multiple layers of eggshell, compacted and disturbed by sediments. The two-dimensionally preserved egg reveals between four and six layers in cross-section (**a**). Parts of the cuticle (cu, dark brown layer) and shell membrane (sm) are also preserved (**b**). A close-up of the shell membrane (pink box) shows some fibrils (pink arrows), that are proteinaceous in living birds, but in this case might be at least partially mineralized. The eggshell layers are highly diagenetically altered (**c**, **d**). A four-layered area with a mirror-image pattern most likely represents an abnormal double-layered egg (**d**, **e**) that displays the tripartite ornithoid microstructure typically found in avian eggshells: a mammillary layer (ml) with organic cores (white arrows), a prismatic layer (pl), and an external layer (el). However, this mirror-image pattern (**e**) could also be the product of sediment displacement and lithostatic compaction, as exemplified in a second ground section (**f**, **g**). In this section (**g**), sediment displacement and shear have also partially created a mirror-image pattern, mimicking an abnormal multi-layered eggshell (**f**). Scale bar is 500 μm in **a** and **g**; 50 μm in **b**, 100 μm in **c**, **d** and **f**; and 20 μm in **e**

A two-dimensionally flattened egg is preserved, laterally bound by the left and right pubes, extending caudally from the penultimate thoracic vertebra and truncating just cranial to the pygostyle (Fig. 1; Supplementary Figs. 1–2). The egg occupies an area measuring approximately 5.2 cm^2^ (Fig. 1) with a thickness between 200 and 400 µm (Fig. 2). Two petrographic ground-sections reveal between four and six compacted layers of eggshell, highly diagenetically altered by dissolution and recrystallization (Fig. 2). EDS indicates the eggshell has been dolomitized, so that it now contains a relatively high amount of magnesium (Supplementary Note 2). Dolomitization is not uncommon in fossil eggshells^16^. The eggshell layers are adjacent (closely adhering) in some regions (Fig. 2d) and separated by sediments in others (Fig. 2c, f–g). The general topography and organization of the eggshell layers, the numerous breakages, and the presence of sediment between layers suggests they have undergone substantial post-mortem displacement and shear as the egg was collapsed into a two-dimensional structure (Fig. 2f).

The first ground section (Fig. 2a) reveals four distinct layers of eggshell that are each approximately 50 µm thick, with the top two layers a mirror-image of the bottom two layers (Fig. 2d, e). The eggshell displays a distinctly avian microstructure^17^ consisting of three sub-layers: a mammillary layer with very few visible organic cores, a prismatic layer, and a very thin external layer (Fig. 2e). This tripartite organization is clearest in the two inner eggshell layers, whereas the outer eggshell layers lack visible organic cores (Fig. 2d, e). The eggshell preserved in IVPP V25371 is thinner than any previously reported enantiornithine eggshell^17^. The continuous layer (prismatic and external layers combined) is thinner than the mammillary layer (cl:ml ratio = 0.58:1) whereas in previously reported enantiornithines^17^ and neornithines^18^, the continuous layer is thicker, typically twice the thickness of the mammillary layer (e.g., cl:ml ratio of 2:1; or 1.9:1)^17^.

**Fig. 3:**
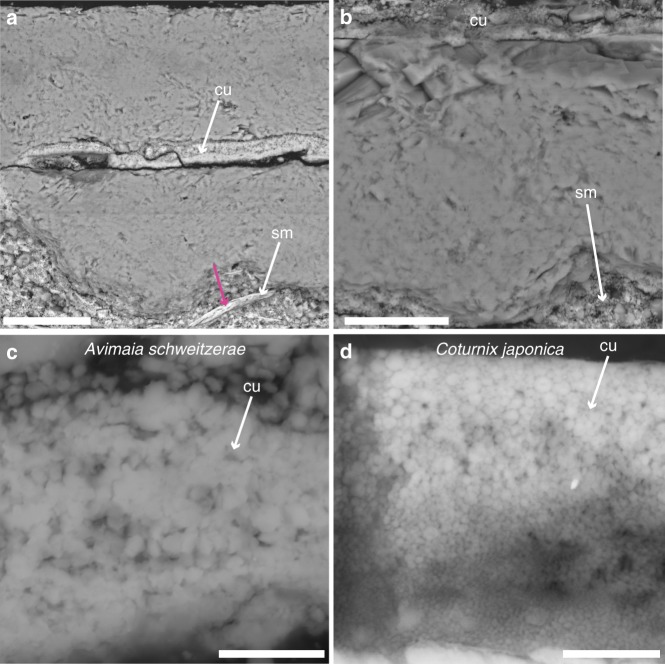
SEM images showing the cuticle, eggshell, and shell membrane of the egg of *Avimaia*. Eggshell, cuticle, and shell membrane in cross-section (**a**). One fibril from the shell membrane is clearly visible (pink arrow). Close-up of the eggshell (**b**) and parts of the overlying cuticle and shell membrane in cross-section (**b**). Close-up of the cuticle of IVPP V25371, showing nanostructures of calcium phosphate (**c**), an adaptation found in modern birds, such as the Japanese quail, *Coturnix japonica* (**d**), to protect the embryo in infection-prone environments. It also represents the hypothesized ancestral morphological condition for avian cuticles. Abbreviations: same as Fig. 2. Scale bar is 50 μm in **a**, 20 μm in **b**, 3 μm in **c**, and 2 μm in **d**

An additional dark brown layer is preserved in some regions (Fig. 2b). SEM and EDS indicate this layer is enriched in phosphorus compared to the rest of the eggshell, suggesting this represents part of the eggshell cuticle (Fig. 3a–c) (Supplementary Note 2), which is high in phosphorous content in extant birds^19,20^. EDS of the surrounding sediment confirms that this enrichment in phosphorus is endogenous to the cuticle layer and is not a contaminant (Supplementary Note 2). SEM also further indicates this layer consists of spherical nanostructures of calcium phosphate (Fig. 3c, Supplementary Note 2). Similar nanospheres are present in the cuticle of some extant birds, such as the Japanese quail (Fig. 3d), although within Neornithes cuticle nanostructures occur in a variety of shapes and sizes^21,22^.

**Fig. 4:**
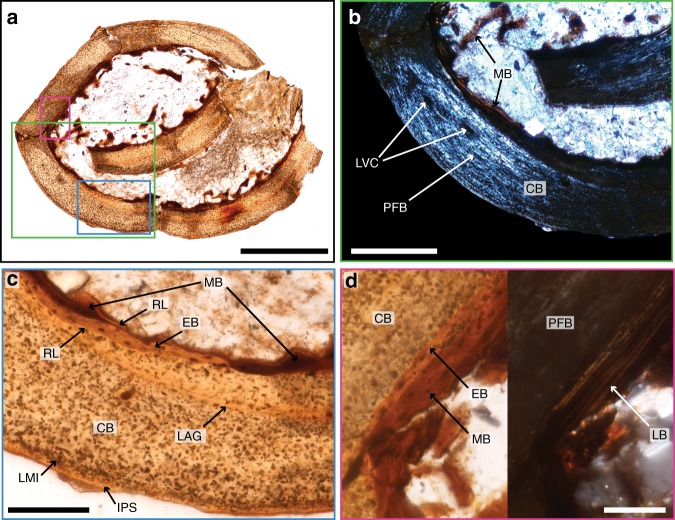
Histology reveals the presence of small amounts of medullary bone (MB) in the femur of *Avimaia*. Natural light (**a**, **c**, **d**—left image) and polarized light microscopy (**b**, **d**—right image) show slow growing, parallel-fibered and almost avascular cortical bone (CB), and small, endosteal trabeculae of lamellar MB (which are much darker than the CB and the endosteal bone, EB) extending into the medullary cavity. Other abbreviations: IPS intact periosteal surface, LAG line of arrested growth, LB lamellar bone, LMI layer of microbial invasion, LVC longitudinal vascular canal, PFB parallel-fibered bone, RL resorption line. Scale bar is 500 μm in **a**, 200 μm in **b**, 100 μm in **c**, and 50 μm in **d**

Parts of what appears to be the organic shell membrane are also visible in both the petrographic slides (Fig. 2b) and SEM images (Fig. 3a). In neornithines, the shell membrane (also called the Membrana Testacea) consists of proteinaceous fibrils of various sizes^23^. In *Avimaia*, these fibrils are between ~1.5 μm (Fig. 2b) and 4 μm thick (Fig. 3a). Both their size and morphology are within the range of those found in modern avian shell membrane^23^ and similar to the permineralized fibers previously reported in other dinosaur eggs, including another fossil avian eggshell from the Late Cretaceous^24–26^. However, EDS analysis of the shell membrane suggests that very few of the original organic components are preserved (Supplementary Note 2).

### Femoral histology

Histological examination of the right femur (Fig. 4) reveals a thin compacta of nearly avascular cortical bone (CB) and the presence of very small amounts of a darker endosteal bone lining the medullary cavity, which we interpret as MB. The compacta consists almost entirely of parallel-fibered bone (Fig. 4b, d). A thin inner circumferential layer (ICL) of endosteally derived lamellar bone separates the MB from the CB (Fig. 4c). Each of these layers is separated by a clear line of resorption (Fig. 4c). Like the eggshell, the CB is heavily altered, displaying irregular and often confluent osteocyte lacunae (Fig.4c–d) typical of post-mortem microbial invasions^27^. The density of these microbial invasions is increased just below the periosteal surface (Fig. 4c), whereas a very thin band of the outermost bone (Fig. 4c) appears almost unaffected by these microbial invasions (Fig. 4c). A similar pattern is very commonly observed in archeological specimens and suggests that the post-mortem microbial invasion stopped before the entirety of the bone was affected^28^. Under normal light the intact periosteal bone external to the microbial invasion may superficially resemble an outer circumferential layer^29^ (OCL), but polarized light analysis reveals no significant differences in birefringence when compared to the rest of the CB (Supplementary Fig. 3).

The CB is poorly vascularized by a very small number of longitudinal vascular canals indicating slow growth. It is interrupted by a single line of arrested growth (Fig. 4c), indicating that this specimen was at least 1-year old. Because no OCL^29^ is present and the ICL is proportionately thin, we infer IVPP V25371 had not reached full skeletal maturity.

At low magnification, the MB consists of a thin, dark brown layer, with some short trabeculae extending into the medullary cavity (Fig. 4). The MB can clearly be distinguished by its color from the lighter colored CB. The endosteal surface of the MB is scalloped, indicating active resorption at the time of death (Fig. 4c). The MB has very few osteocyte lacunae and those present are elongated (Fig. 4d). Polarized light reveals a lamellar organization of the collagen fibers (Fig. 4d).

### Interpretation of preserved eggshell

We interpret the entire preserved eggshell structure as a single egg with an additional, abnormal layer of eggshell, that has been crushed flat and disturbed by sediments during diagenesis. Although multiple layers are preserved, if two eggs were present the top two eggshell layers should mirror each other, as should the bottom two layers. Instead the top two layers mirror the bottom two layers (Fig. 2e). Although only one small fragment of the egg was histologically sampled, the entire structure was analyzed through CT and the data show no indication that a second egg was present (Supplementary Fig. 2). Our interpretation that only a single egg is present is consistent with evidence from preserved soft-tissue and nests that suggest stem birds (and possibly troodontids) had only a single functional ovary and would have laid one egg per day^7^.

Four closely adhering layers of eggshell with the top two layers mirroring the bottom two layers (Fig. 2d, e) are not apparent throughout the entirety of the ground-sections (Fig. 2c, f, g). In some areas only two mirrored layers are visible with additional layers present but separated by matrix and not forming a clearly double-layered (on top and bottom) structure (Fig. 2c). There are two possible interpretations: either the second layer of eggshell detached from the inner layer during diagenesis in some regions (and was lost in some areas), which frequently occurs in the extra layers of modern bird eggs when this abnormality is reported^30,31^, or the four-layered, mirror-image pattern observed in the first section (Fig. 2d, e) is the product of complex lithostatic compaction, post-mortem folding, and sediment displacement of a single-layered eggshell. It is often difficult to make this differentiation in fossils^32,33^. In the second ground section (Fig. 2f, g), the bottom-most layer has clearly been displaced and sheared and in fact belongs to the layer now located above it (Fig. 2f). This exemplifies how sediment displacement can create a mirror-image pattern mimicking a multilayered egg.

Additional CT-scan data with extremely high-resolution capable of reconstructing the layers throughout the entire egg may be able to distinguish between these two hypotheses in the future. However, three lines of evidence indicate that at this time, the preserved structure is best interpreted as an abnormal, double-shelled egg that has been somewhat obscured by diagenesis. First, the observed eggshell layers are not morphologically consistent throughout: the mammillary layer in the outer layers of eggshell lacks visible organic cores and it appears only the inner layer has cuticle (Fig. 2d, e). If the layers are all from a single-layered egg this difference in morphology between inner and outer layers should not exist. Secondly, the absence of visible organic cores only in the outer layers strongly suggests that fewer nucleation sites were available during the formation of the outer eggshell layer. This often occurs in the outer layers of abnormal multilayered non-avian dinosaur eggs^34^. Finally, the proportions of the layers making up the eggshell microstructure are atypical further supporting inferences that the reproductive system in this individual was disrupted. In all healthy neornithine eggs^18,35^ and in previously reported enantiornithine eggs^17^, the continuous layer is thicker than the mammillary layer (typically cl:ml ratio = 2:1), whereas the opposite condition is present in IVPP V25371 (cl:ml ratio = 0.58:1). The presence of a cuticle (Figs. 2b, 3) indicates that the formation of the continuous layer had terminated and that its unusually thin morphology is not because it was incomplete. This strongly suggests that the reproductive system of *Avimaia* IVPP V25371 was not functioning normally, supporting interpretations regarding additional egg abnormalities.

## Discussion

**Fig. 5:**
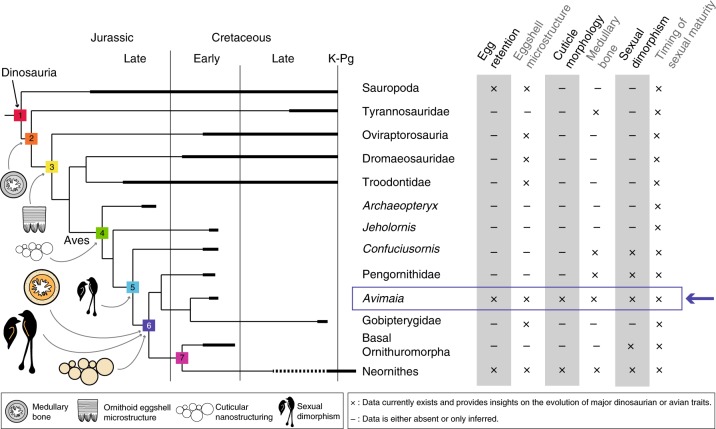
Simplified phylogeny with hypothetical evolutionary stages of modern avian reproduction. Stage 1: Plesiomorphic dinosaurian condition: abnormal multilayered eggshells due to egg retention can occur^34^, dinosauroid eggshell microstructure^35^, possible sexual dimorphism^75^, strong histological evidence for sexual maturity occurring prior to skeletal maturity^63^. Stage 2: Possible appearance of medullary bone^4,8^. Stage 3: Possible appearance of ornithoid eggshell microstructure^35^. Stage 4: Possible appearance of cuticular nanostructuring^21^. Stage 5: Strong evidence for sexual dimorphism (inferred sexually dimorphic feathers, e.g., ref. ^65^). Stage 6: In *Avimaia*: presence (not necessarily first occurrence) of cuticular nanostructuring, medullary bone, and a plesiomorphic non-avian dinosaur-like timing of sexual maturity within Aves. Further support for the presence of sexually dimorphic plumage. Stage 7: Avian-like timing of sexual maturity, skeletal maturity occurs prior to sexual maturity (probably evolved multiple times in parallel in Ornithuromorpha^64^)

Reports of dinosaur specimens preserved with eggs, or other key reproductive features such as ovarian follicles, preserved within the body cavity, are extremely rare^7,36,37^. The holotype of *A. schweitzerae* is the only fossil bird ever reported with an unlaid, intra-abdominal egg. The total evidence from the preserved eggshell microstructure combined with histological data inform on a number of important evolutionary questions pertaining to the origins of the specialized form of reproduction present in modern birds (Fig. 5).

The eggshell preserved in IVPP V25371 is abnormally thin, as indicated by the unusual proportions of the continuous layer relative to the mammillary layer. Eggshell thinning in extant birds is often attributed to environmental pollutants^38^ but can also be caused by a hormonal imbalance^39^. The egg is also best interpreted as having two layers of eggshell (Fig. 2e). Multilayered (including double-layered) eggs are common in non-avian dinosaurs and in both fossil and extant turtles, and are commonly referred in the literature as pathological or abnormal^31,32,34,40^. Because these types of eggs have been described in fossils more extensively than in extant species, more data are necessary to fully understand the cause of these numerous abnormalities preserved in the fossil record^41^. However, multilayered eggs clearly do not represent the normal, healthy condition. It has been suggested that they represent an ubiquitous response to stress in the many lineages in which they occur^31,34^.

Modern eggs with multiple layers of eggshell are caused by abnormal, prolonged retention in the oviduct, which is often attributed to various types of physiological and/or environmental stresses^31,41–45^. Although this condition is common in non-avian dinosaurs and turtles^31,32,34,40^, multilayered eggshells appear to be comparatively rare in modern birds, to our knowledge only reported in a handful of species (the chicken^44,46,47^, the Herring Gull^48^, the Japanese Quail^31^ and the Tropical mockingbird^30^), and this condition has not been previously reported in any fossil bird. If this is not the product of a sampling bias, it is unclear why this condition would be less common in Neornithes compared to more basal reptiles but it may be related to physiological changes in crownward avians.

In modern birds, the eggshell cuticle reportedly only forms an hour before oviposition^49^. *Avimaia* IVPP V25371 appears to preserve parts of the cuticle layer. If our interpretation is incorrect and the egg is not multilayered, this suggests eggshell formation was completed but the bird died within the hour after cuticle formation before oviposition. However, we infer that the eggshell is double-layered and note that the cuticle only appears to be present on the inner eggshell layer (Figs. 2, 3). This strongly suggests that after eggshell formation was complete (as evidenced by the cuticle), the egg was abnormally retained within the oviduct where it received a second layer of eggshell. This evidence suggesting prolonged egg retention, together with the fact the egg is preserved still inside the body, brings us to speculate that *Avimaia* IVPP V25371 may have suffered from a serious condition known as egg binding (also referred to as egg impaction). This condition, in which the egg becomes completely stuck inside the body, is a form of aggravated egg retention^42,45^. Egg binding is common in small, domestic birds^45,50^, also occurring in the wild^51^, and typically results in death unless the egg is manually or surgically extracted. Thus, it is possible that IVPP V25371 died as a direct result of the abnormal egg preserved inside it. If correct, this would represent the oldest documented occurrence of this common reproductive disorder. The alternative hypothesis, based on the timing of cuticle formation of healthy avian eggs^49^, that *Avimaia* IVPP V25371 died within the hour before oviposition due to causes unrelated to egg retention or binding, while gravid with an abnormal egg, is possible but seems less likely given the multiple lines of evidence indicating dysfunction of the reproductive system.

The egg preserved in *Avimaia* IVPP V25371 also offers insight into the evolution of the avian cuticle. Fossilized cuticles are rarely reported, but remnants are known from non-avian theropod^20^ and enantiornithine^17,52^ eggshells. However, none reported the presence of nanospheres of calcium phosphate (Fig. 3c). In neornithines these nanospheres play multiple roles in the survival of the developing embryo (e.g., Fig. 3d), including critical antimicrobial defense^21,22^. Although most modern birds lack nanospheres in their cuticle, nanostructuring has been hypothesized to represent the ancestral avian condition^21^ (Fig. 5). This hypothesis is supported by the discovery of nanospheres in the cuticle preserved in *Avimaia* (Fig. 3c). Similar nanospheres most commonly occur in modern taxa that nest in humid, infection-prone environments (e.g., nests near water, or buried^21^); therefore, we hypothesize a similar nesting ecology for *Avimaia*. This also corroborates data from preserved enantiornithine nests, which suggests the eggs were partially buried^53^. Future biomolecular analyses, such as investigation of the eggshell pigment, may allow further insights into the nesting ecology of *Avimaia*^54,55^.

Because IVPP V25371 represents a gravid female bird, we anticipated the presence of MB, a labile tissue that forms endosteally in cancellous spaces throughout the skeleton in ovulating and gravid female birds, providing a calcium reservoir for eggshell formation^3,5^ (Fig. 4). MB was first reported outside Neornithes in the non-avian theropod *Tyrannosaurus rex*, indicating this reproductive specialization evolved far outside of Aves^4,8^ (Fig. 5). Since this discovery, MB—or MB-like tissues—have been reported in an even broader phylogenetic sample, including ornithischian dinosaurs and pterosaurs^56,57^. However, because MB-like endosteal tissues can form for a number of reasons not necessarily related to reproduction (e.g., ref. ^58^), no previous report of fossil MB can claim to be unequivocal within the framework of our current understanding of this reproductive tissue^57^. Among stem birds, MB has previously been reported in the fossil bird *Confuciusornis*^59^ and a pengornithid enantiornithine, both from the Jehol Biota^57^. The purported MB in *Confuciusornis* does not hold up to scrutiny and the pengornithid, although a very strong case is made, lacks irrefutable signs of reproductive activity (see discussion in ref. ^57^). IVPP V25371 preserves unequivocal evidence of reproductive activity and lacks indication of bone pathology (i.e., it has a smooth periosteum). Therefore, *Avimaia* IVPP V25371 provides the strongest evidence to date for MB occurring outside crown birds (Fig. 5).

In modern birds, MB is usually woven^4,8^ or parallel-fibered^60^, but it can also present a lamellar organization^61^. Information from this specimen and a previously described enantiornithine^57^ suggests that MB may have formed more slowly in enantiornithines compared to similarly sized neornithines. This is consistent with CB deposition, which is slower in enantiornithines compared to crown birds^62^. The MB in *Avimaia* appears to have formed more slowly than that reported in another Early Cretaceous enantiornithine^57^, which likely represents intraspecific variation. The femoral histology indicates IVPP V25371 was not rapidly growing but also that it had not fully reached skeletal maturity at the time of death. This provides further evidence in support of hypotheses that non-ornithurine birds, like non-avian dinosaurs but unlike extant birds, reached reproductive maturity before somatic maturity^59,63–65^ (Fig. 5). This has been demonstrated in non-avian dinosaurs based on specimens preserved while brooding^66^. Although widely speculated to also be true in non-ornithurine birds (and in some cases supported by purported occurrences of MB)^56,59^, this specimen provides additional evidence that reproductive maturity preceded skeletal maturity in stem birds.

Lastly, the holotype of *Avimaia* appears to lack elongated tail feathers (Fig. 1), which lends support to inferences regarding the sexual dimorphism of elongate tail feathers in enantiornithines (Fig. 5). Some specimens of confuciusornithiforms and enantiornithines preserve elongate rachis-dominated tail feathers (RDFs), which are widely considered to be sexually dimorphic features and present only in males^65,67^. Support for this interpretation comes from the purported preservation of ovarian follicles in one enantiornithine^6,7^ and medullary bone in one *Confuciusornis*^59^, but the identification of female specific tissue in both cases is still controversial^57,68^. Consistent with preserved indicators that IVPP V25371 is female (egg, MB), RDFs are absent. It is possible the absence of RDFs in IVPP V25371 is preservational (as is the case in all described specimens lacking this feature), given that feathers are not well preserved in this specimen. An absence due to seasonal differences in plumage is unlikely—if these feathers are in fact a sexual characteristic, they would be expected to be present either year-round (as suggested by their presence in very young juveniles^69^) or only present in the breeding plumage. Since IVPP V25371 was clearly breeding, the absence of RDFs in this specimen is most likely either a true absence or taphonomic artifact. If this is a true absence, the holotype of *Avimaia* then also supports previous hypotheses that such features are sexually dimorphic and only present in male enantiornithines^67^.

## Methods

### Phylogenetic analysis

We explored the phylogenetic position of IVPP V25371 (Supplementary Figure 4) using an expanded version of the O’Connor and Zhou^70^ dataset^15^ that includes seven additional tarsometatarsal characters (Supplementary Note 1) and revised cranial scorings for *Ichthyornis* based on recently published data^71^. We included all previously described enantiornithines from the Xiagou Formation: *Qiliania, Feitianius, Dunhuangia*, GSGM CAGS-IG-07-CM-001, CAGS-IG-02-0901, CAGS-04-CM-023. Information from CAGS-04-CM-007 was combined with IVPP V25371 for the operational taxonomic unit (OTU) *Avimaia*. CAGS-04-CM-023^72^ had to be removed because inclusion of this specimen resulted in the collapse of Ornithothoraces. The final matrix consists of 41 taxa, 25 of which are enantiornithines, scored across 252 characters, 31 of which are ordered, which we analyzed using TNT^73^. Early avian evolution is extremely homoplastic; thus, we explored the effects on the matrix of using implied weighting at various *k* values (*k* = 1, 3, 12, 16)^74^. We found that the results using a priori weights were identical to those using implied weighting with the recommended *k* value of 16^74^. Lower *k* values created a slightly larger polytomy among derived enantiornithines.

In the presented analysis we conducted a heuristic search using tree-bisection reconnection (TBR) retaining the single shortest tree from every 1000 followed by a second round of TBR. The first round produced two trees 709 steps long; a second round of TBR produced 44 trees of the same length.

### Surface area calculations

The surface area occupied by the flattened egg was calculated using a scaled photograph into the software Illustrator CS6 and the area-length plug-in.

### Paleohistology—petrographic ground-sections

The two fragments (from the eggshell and the femur, Supplementary Figure 2) were extracted with a rotating tool equipped with a diamond blade (Dremel 8100). Subsequently, the fragments were prepared using the paleohistological ground-sectioning technique: Samples were embedded in EXAKT Technovit 7200 (Norderstedt, Germany) one-component resin, and cured for 24 h, cut using an EXAKT 300CP accurate circular saw, and then ground and polished using the EXAKT 400CS grinding system (Norderstedt, Germany) until the desired optical contrast was reached (between 20 and 30 μm). Sections were observed under both natural and elliptically polarized light using a ZEISS AX10 light microscope (Thornwood, USA). Photographs were taken using the camera ZEISS AxioCam MRc5 (Thornwood, USA) and the software Axio Vision SE64 (Rel. 4.9). We used the “photomerge” tool in Adobe Photoshop CS6 to reconstruct the entire sections.

### SEM and EDS

SEM was taken at Chinese Academy of Geological Sciences using FEI Quanta 450 (FEG) at 20 kv. For the fossil egg, petrographic ground-sections were used, but for the extant comparison of the Japanese quail cuticle (*Coturnix japonica;* Fig. 3d), we used a fragment of eggshell embedded in a polished, resin block (EXAKT Technovit 7200 (Norderstedt, Germany). The Japanese quail egg fragment was taken from a complete egg obtained commercially. This species was specifically chosen for its cuticle morphology (with nanostructures of calcium phosphate) based on two previous studies^21,22^. Both BSE and SE modes (backscattered electrons and secondary electrons) were applied and EDS was calculated for several spots (Supplementary Figure 5) showing different structures (Supplementary Note 2, Supplementary Figs. 5–11, Supplementary Table 1).

### μComputed tomography

The egg of IVPP V25371 was scanned twice. The longitudinal scan (with a resolution of 15.49 μm) (Fig. 2b; Supplementary Figure 2b) was obtained from a 160-Micro-CL (Computed Laminography) at the IVPP (Beijing China). The cross-sectional CT scans (15.7 μm) (Supplementary Figure 2c) were obtained from an industrial CT scanner Phoenix v|tome|x (Shanghai, China). CT scans were observed and photographs were taken using the software VGSTUDIO MAX (2.0).

### Nomenclatural acts

This published work and the nomenclatural acts it contains have been registered in ZooBank, the proposed online registration system for the International Code of Zoological Nomenclature (ICZN). The ZooBank LSIDs (Life Science Identifiers) can be resolved and the associated information viewed through any standard web browser by appending the LSID to the prefix “http://zoobank.org/”. The LSIDs for this publication are: urn:lsid:zoobank.org:act:66CA3084-07B6-47F9-8F8D-3CC27C72678D.

### Reporting summary

Further information on experimental design is available in the Nature Research Reporting Summary linked to this article.

## Supplementary information

Supplementary Information

Reporting Summary

## Data Availability

IVPP V25371 is reposited at the Institute of Vertebrate Paleontology and Paleoanthropology in Beijing. All data are available upon reasonable request.
