# Supplementary material for: An Early Cretaceous enantiornithine (Aves) preserving an unlaid egg and probable medullary bone
Source: Nat Commun. 2019 Mar 20;10:1275. doi: 10.1038/s41467-019-09259-x (PMC6426974; doi:10.1038/s41467-019-09259-x)
Supplement: Supplementary file 1 — Supplementary Information [file 41467_2019_9259_MOESM1_ESM.pdf]

Supplementary Information

An Early Cretaceous enantiornithine (Aves) preserving an unlaid egg and probable medullary bone

Bailleul et al.

This PDF file includes:

Supplementary Notes

Supplementary Note 1: Additional Methods, and phylogenetic scorings and characters

Supplementary Note 2: EDS (Energy Dispersive Spectroscopy) Analysis

Supplementary Figures 1 to 11

Supplementary Table 1

Supplementary References

Supplementary Note 1: Additional Methods, and phylogenetic scorings and characters

The phylogenetic trees resulting from the analysis only differed in the relative placement of some derived enantiornithines. The results largely agree with recent analyses. In the strict consensus tree (Consistency Index: 0.465; Retention Index: 0.626) *Avimaia* forms part of a derived polytomy that includes *Neuquenornis, Enantiophoenix, Concornis, Eoenantiornis*, and GSGM-07-CM-001 (Supplementary Fig. 4). This polytomy forms a dichotomy with a clade formed by *Feitianius* + *Eoalulavis* and *Cathayornis yandica*. This larger clade forms a polytomy with *Elsornis, Qiliania, Halimornis,* and *Shenqiornis*. These taxa form a dichotomy with the Longipterygidae, which consists of a polytomy between *Longirostravis, Rapaxavis*, and *Shanweiniao* with *Longipteryx* falling just outside this polytomy. *Dunhuangia* + CAGS-IG-02-0901, *Eocathayornis, Protopteryx, Eopengornis* + *Pengornis*, and *Iberomesornis* + *Gobipteryx* form successive outgroups. The basal position of *Gobipteryx* (typically resolved fairly derived in the tree) in this analysis is unusual. However, it is worth nothing that most Xiagou enantiornithines are resolved fairly derived in the tree, supporting hypotheses that this fauna is younger and more advanced than the Jehol avifauna.

Scorings:

Avimaia IVPP V25371 + CAGS-04-CM-007: 0 ?????????? ?????????? ?????????? ?????????? ?????????? ??????0??1 [01]??[123]????24 ????11??? ?????????? ?????????? ?????????? ?????????? ?????????? ?????????? ?????????? ?????????? ?????????? ????????00 0?0??1?110 1?11?0[12]1?? 111010??10 1010?01??? 1001001?01 0220020000 011?000100 11

Character changes (see also Atterholt et al.[1])

Modified Character 233 (state 3 new)

Metatarsal II tubercle (associated with the insertion of the tendon of the m. tibialis cranialis in Aves): absent (0); present, on the medial dorsomedial margin of metatarsal II (1); located approximately the center of the proximodorsal surface of metatarsal II (2);

developed on lateral surface of metatarsal II, at contact with metatarsal III or on lateral edge of metatarsal III (3). (ORDERED)

New Characters 246 – 252:

246. Metatarsals II and III, tubercle for muscle attachment (presumably the m. tibialis cranialis): one such tubercle (0); two tubercles present (1). (modified from O'Connor et al., 2014[2]).

247. Position of the tubercle for the m. tibialis cranialis: proximally located (0); located approximately 1/3 from the proximal end (1); located near to or distal from the midpoint (2). (from O'Connor, 2009[3])

248. Tubercle for the m. tibialis cranialis hypertrophied: absent (0); present (1). (New)

249. Medial condyle of metatarsal III trochlea projecting strongly plantarly relative to the lateral condyle: absent (0); present (1); lateral condyle projects farther (2). (modified from Chiappe, 1993[4]).

250. Medial trochlea of metatarsal III, protrudes farther distally than lateral trochlea: absent (0); present (1). (New)

251. Dorsal surface of metatarsal III strongly convex: absent (0); present (1). (from Chiappe, 1993[4])

252. Metatarsal IV trochlea: ginglymous (0); reduced to a single convex surface in caudal view (1); single condyle medially excavated (2). (modified from Chiappe, 1993[4]).

Synapomorphies:

107: 3 -> 2 (hypocleidium 30% length of furcular rami; ambiguous)

188: 0 ->1 (ischium more than 2/3 length of the pubes; ambiguous)

236: 1->2 (metatarsal II shorter than metatarsal IV, reaching distally only as far as base of metatarsal IV trochlea; ambiguous)
251: 0->1 (Dorsal surface of metatarsal III strongly convex; ambiguous)

CAGS-IG: Chinese Academy of Geological Sciences, Institute of Geology, in Beijing, China; FDRC: Fossil Research and Development Center, of the Third Geology and Mineral Resources Exploration Academy, Gansu Provincial Bureau of Geo-Exploration and Mineral Development, Lanzhou, China.

Although never formerly described, IVPP V25371 was included in phylogenetic analyses informally referred to as “CAGS-IG-06-CM-012” by O’Connor (2009)[3] and later as “FDRC-06-CM-012” You et al[5] and Ji et al[6]. The number “06-CM-012” refers to its field catalogue number (06 signifying it was collected in 2006, CM representing the locality ChangMa, and 012 referring to the fact it was the 12th catalogued specimen). This is the first time this specimen has been formally assigned a collection number and placed in a repository, and the specimen should be officially referred to as IVPP V25371.

Supplementary Note 2: EDS Analysis of the eggshell, remnants of the cuticle, surrounding sediment, and fragments of the shell membrane, and a discussion regarding identification of the cuticle.

General observations:

EDS spectra where taken at different spots on a ground-section observed under SEM (Supplementary Fig. 5). Spots 1 and 2 are made in the eggshell (Supplementary Figs. 6-7); Spots 3 and 4 in the cuticle (Supplementary Figs. 8-9), spot 5 in the sediment (Supplementary Fig. 10, and spot 6 in the organic shell membrane (Supplementary Fig. 11). The two eggshell results (spots 1 and 2; Supplementary Figs. 6-7) have very similar proportions for the major elements found (Ca, Mg, O, Fe); the cuticle differs clearly from the eggshell, with a higher proportion of P, but is lower in O and Fe (Supplementary Figs. 8-9). The shell membrane is distinct in being enriched in Si, O and Fe (Supplementary Fig. 11)

Cuticle identification:

An enrichment in P has been determined to be a marker of the cuticle, if the surrounding sediment is depleted in P, and if it can be shown that the enrichment in P is not a contamination from the sediment[7]. First of all, there is no sediment directly in contact with the cuticle layer in any area where it is preserved. It is only found where the two eggshell layers are preserved adjacent, in close contact (which may have facilitated preservation of the cuticle), therefore a contamination seems unlikely based solely on a topographical perspective.

Second, EDS analyses further suggest that the higher concentration in P in the cuticle when compared to that of the rest of the eggshell (compare Supplementary Figs 8-9 to Supplementary Figs. 6-7), does not come from a phosphorous contamination from the sediment (Supplementary Fig. 10). Supplementary Table 1 compares P, Ca and Si in these three different types of sampled material: the sediment has a high proportion of Ca because the Changma locality is mostly composed of lacustrine carbonates[8]. There is a relatively high proportion of P when compared to that of the eggshell (10.96% in the sediment, versus 0.56% in the eggshell, see Supplementary Table 1), but the Ca/Si ratios

in the cuticle and sediments are extremely different: i.e., the Ca/Si ratio in the cuticle layer is much higher (78.3%) than that of the sediment (5.18%). Since their composition is so different, it is highly unlikely that the increase in P found in the cuticle is due to contamination from the sediment. If this were the case, a more similar Ca/Si ratio would be expected between these two layers (i.e., an enrichment of the cuticle in Si from the sediment as well as an enrichment in P). This confirms the endogeneity of the P enrichment in the cuticle in comparison to the rest of the eggshell.

Moreover, the nanostructures of Calcium phosphate observed in the cuticle of *Avimaia* (Fig. 3c) are comparable to those found in the cuticle of modern birds, in both their morphology and their chemistry[9,10] (Fig. 3d). All of these arguments (based on topography, morphology and chemistry) strongly suggest that the layer in question has been correctly identified as the cuticle.

Shell membrane:

As mentioned above, the shell membrane, or membrane testacea is distinct in being enriched in Si, O and Fe (Supplementary Fig. 11). Si is also found in extant shell membranes[11] but it is still unclear if this enrichment in Si is a residue of the original shell membrane or if it is derived from bacteria, facilitating the fossilization of shell membrane fibres[12]. Because there is no notable peak of carbon in the spectra from the shell membrane (Supplementary Fig. 11), this suggests that even though the morphology of these fibres is well preserved, very little of the original organic material is left (e.g., compare with results of Soledad-Fernandez and Salgado[11], and Schweitzer and colleagues[13] who do find an enrichment in carbon in the shell membrane when compared to the eggshell in the fossilized avian eggs and sauropod eggs they sampled).

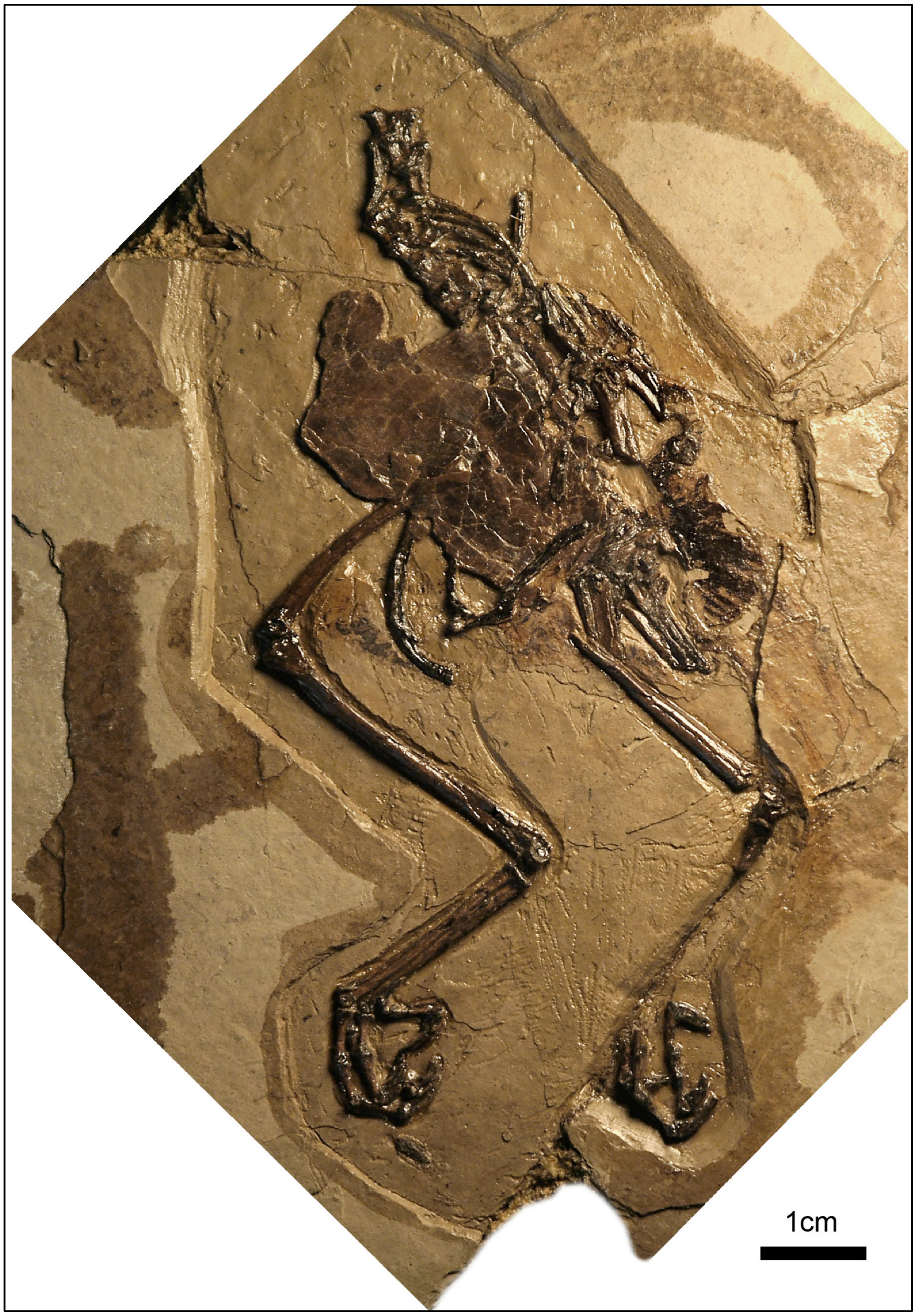

Supplementary Figure 1. Photograph of the new specimen of *Avimaia schweitzerae* (IVPP V25371) prior to any fragment extraction.

This specimen consists of an articulated partial skeleton, feather impressions and intra-abdominal egg.

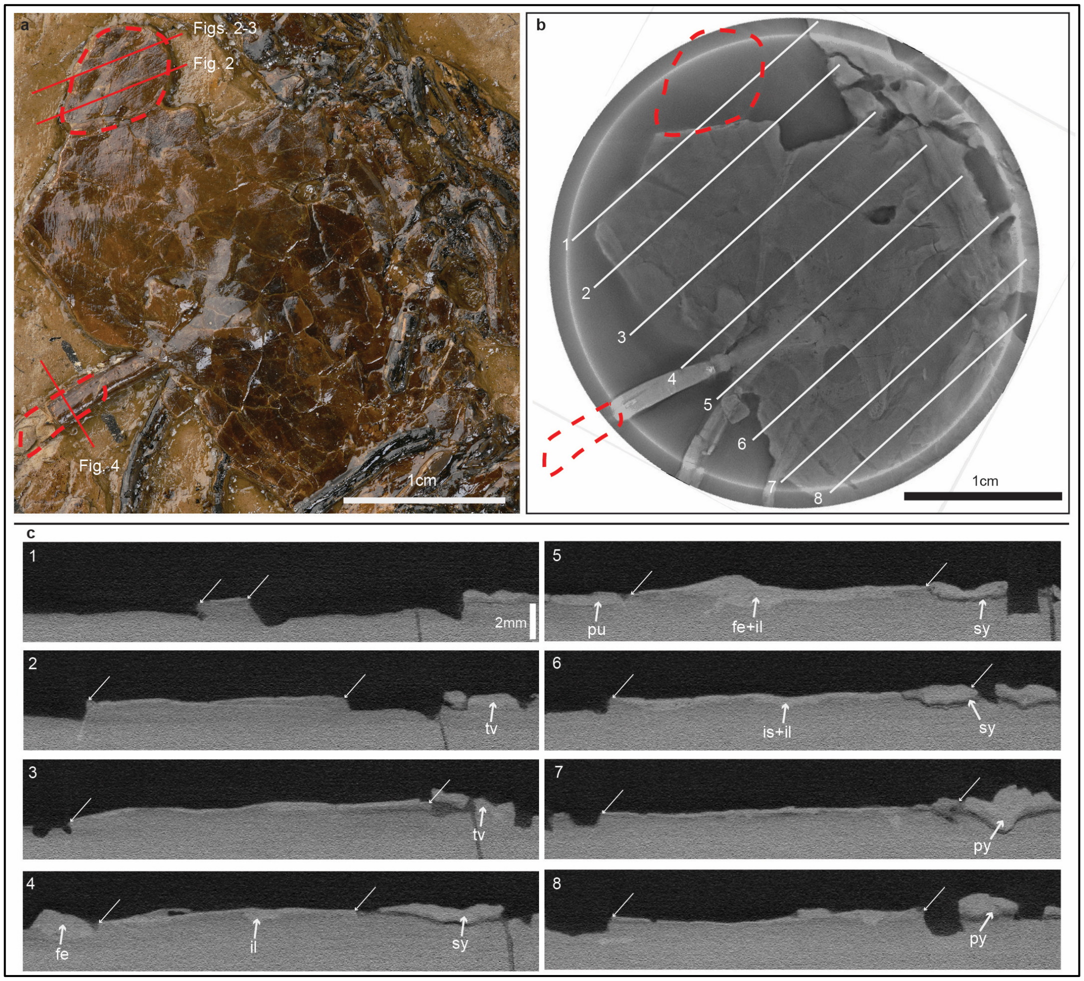

Supplementary Figure 2: Gross morphology of the crushed egg in IVPP V25371 showing the location of the two extracted fragments for petrographic sectioning (a), longitudinal µCT scan (b) and cross-sectional µCT scans (c).
The solid red lines in **(a)** indicate were the two ground-sections of the egg come from (shown in Figs. 2-3), and where the one from the femur was taken (shown in Fig. 4). The extracted fragments for microscopic analyses are shown by the red dashed lines. A microCT scan (15.49µm) of the entire egg after extraction of the fragments shows the pelvic girdle beneath it (**b**). White lines indicate the areas shown with cross-sectional µCT scans (15.7µm) in (**c**). Cross-sectional CT-scans show the overall morphology of the crushed egg and bones beneath it. White arrows indicate the limits of the egg. These CT scans show the overall architecture of the flattened egg in the areas that were not serially sectioned, but even at this high-resolution, accurate eggshell thickness measurements cannot be made. However, the thickness appears to be the roughly the same throughout the structure, without any area showing a significant increase (except when bones are found underneath it). Because the thickness appears roughly the same throughout the structure, this suggest that indeed, only one egg is present within this specimen, and it is not an assemblage of more than one egg. Abbreviations: fe, femur; il, ilium; is, ischium, pu, pubis; py, pygostyle; tv, thoracic vertebra, sy, synsacrum.

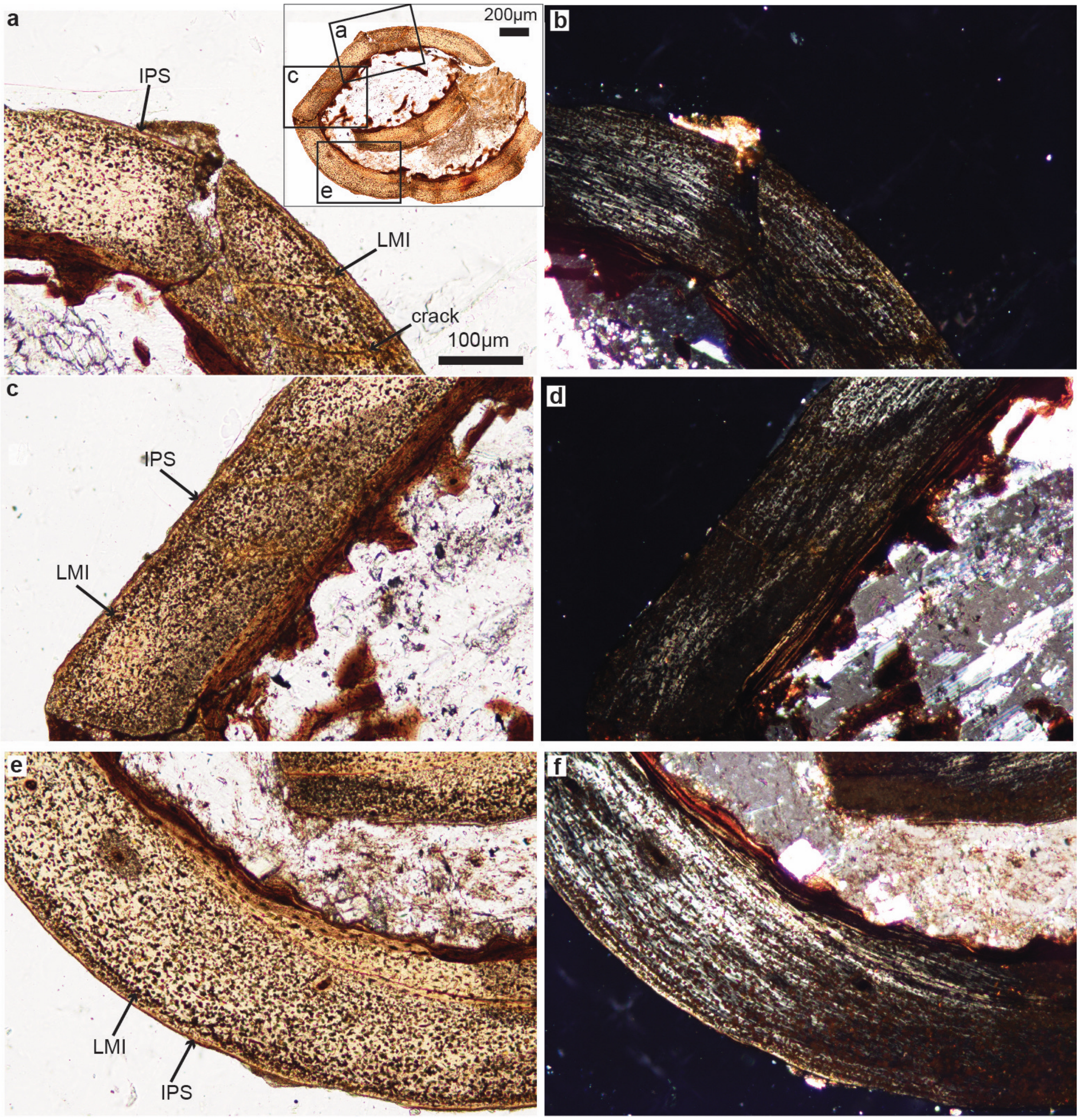

Supplementary Figure 3. Additional histological images of the periosteal surface of the femur of *A. schweitzerae* in natural (a,c,e) and polarized light (b,d,f).
The histological structures of this specimen have suffered significant post-mortem alterations. Two main layers can be identified nearing the periosteal surface: an area with an increased density of microbial invasions (layer of microbial invasions, LMI), and more externally, a thin band of intact periosteal surface (IPS), unaffected (or much less affected) by microbial invasions. Even though it is counterintuitive, it is not uncommon for microbial invasions to stop before reaching the periosteal surface (see Figures 10.4 and 10.6 in Fernandez-Jalvo and colleagues[14]).
These two layers (LMI and IPS) may be misinterpreted as an Outer Circumferential Layer (OCL): in one area they do appear to show a slight difference in collagen fiber orientation when compared to that of rest of the cortical bone (e-f), but this is most likely attributed to the very intense postmortem alteration in the LMI in this area, and most importantly, such a difference (between the cortical bone and the LMI and IPS together) is not seen in the rest of the section (a-d). In modern birds, OCLs are continuous throughout the entirety of the periosteal surface[15]. The IPS, LMI and cortical bone are all parallel-fibered, and the LMI and IPS do not show any significant change in collagen fiber orientation under polarized light (a-d), which do not suggest any significant slow-down of growth, indicating this female bird had not yet reached skeletal maturity at the time of death. All images are at the same scale (same as in a).

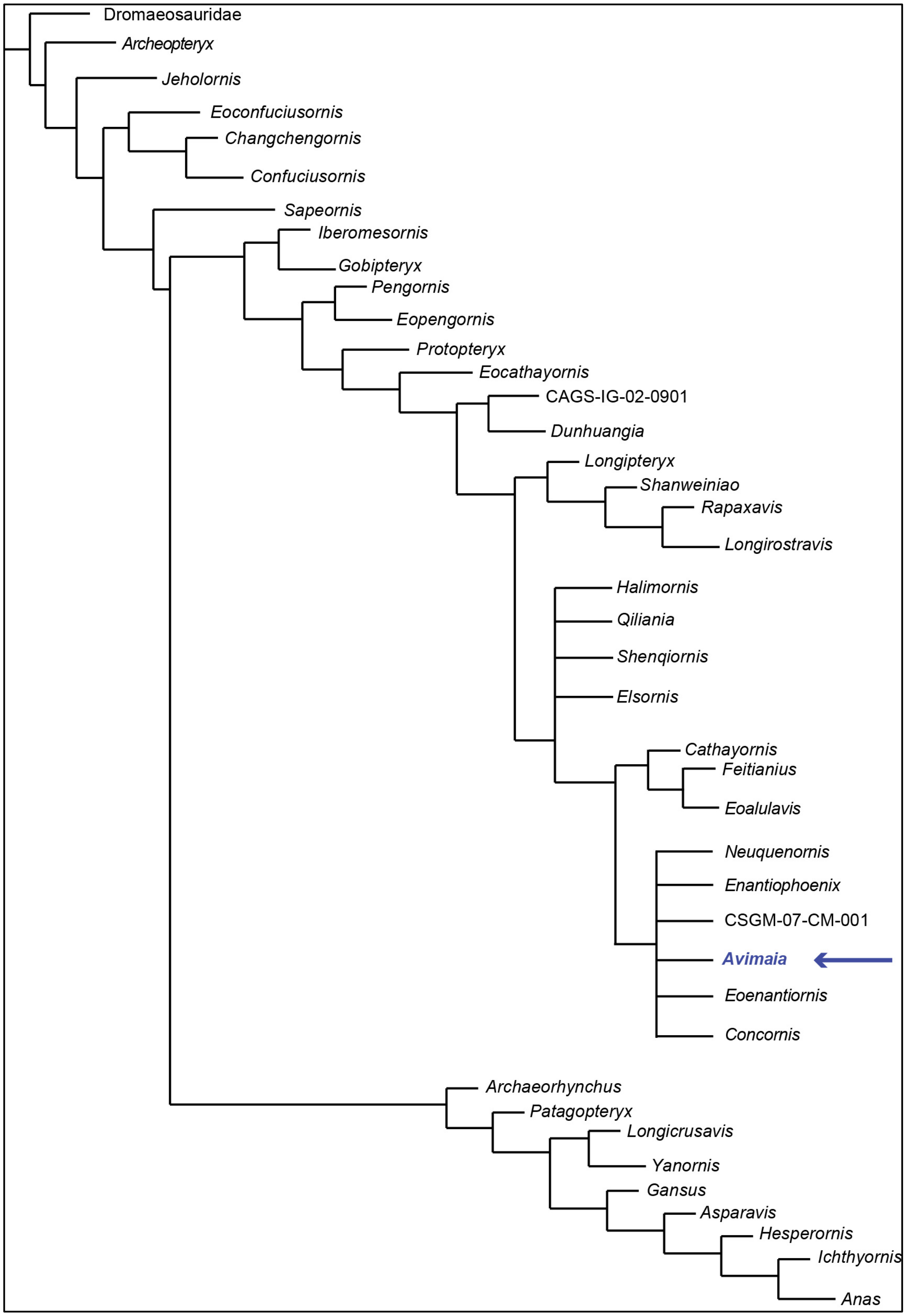

Supplementary Figure 4. Strict consensus of 44 trees depicting current hypothesis regarding phylogenetic position of *Avimaia schweitzerae*, gen. et sp. nov. *Avimaia* is placed within enantiornithines (L = 709; Consistency Index = 0.465; Retention Index = 0.626).

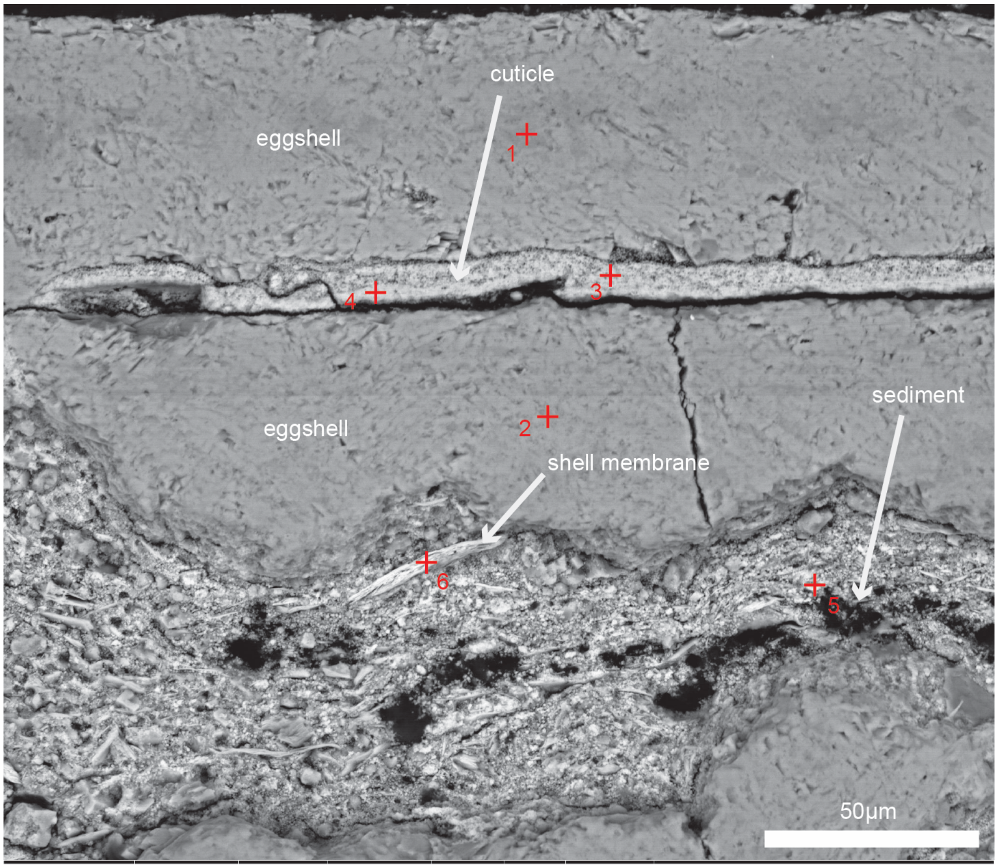

Supplementary Figure 5. SEM section showing areas where the EDS spectra were taken (including the area seen in Fig. 3a in the main manuscript). Spots 1 and 2 are made in the eggshell; Spots 3 and 4 in the cuticle, spot 5 in the sediment, and spot 6 in the organic shell membrane).

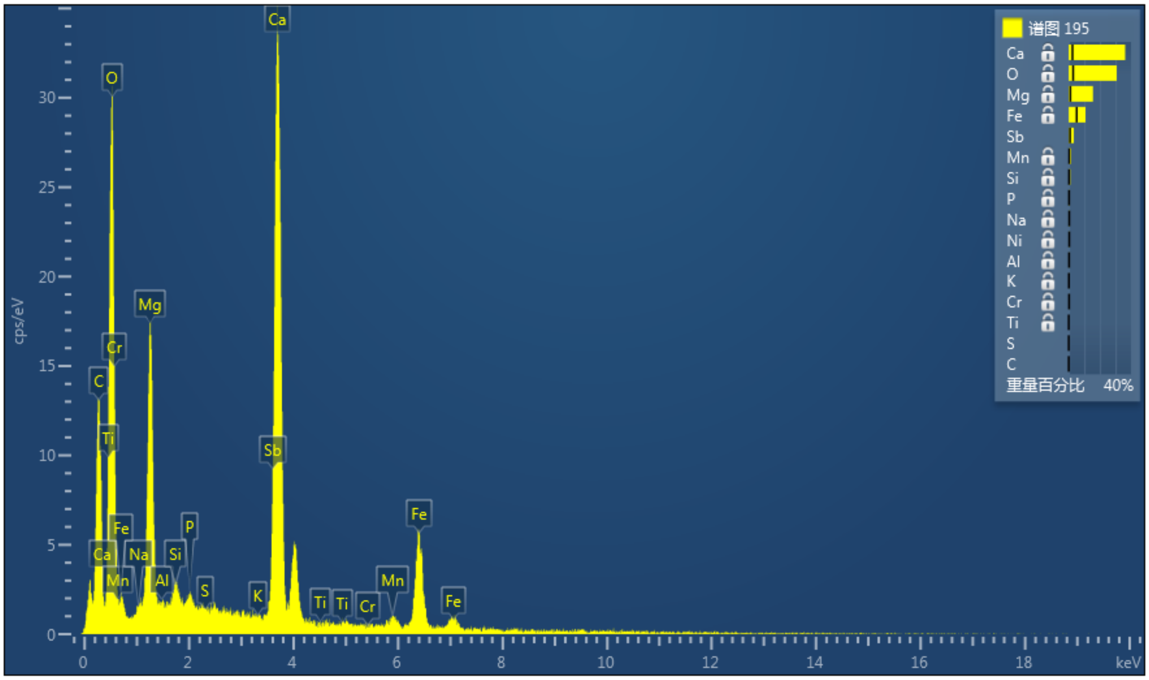

Supplementary Figure 6. EDS spectrum taken into the eggshell at Spot 1.

This spectrum shows an enrichment in Ca, Mg, O, and Fe.

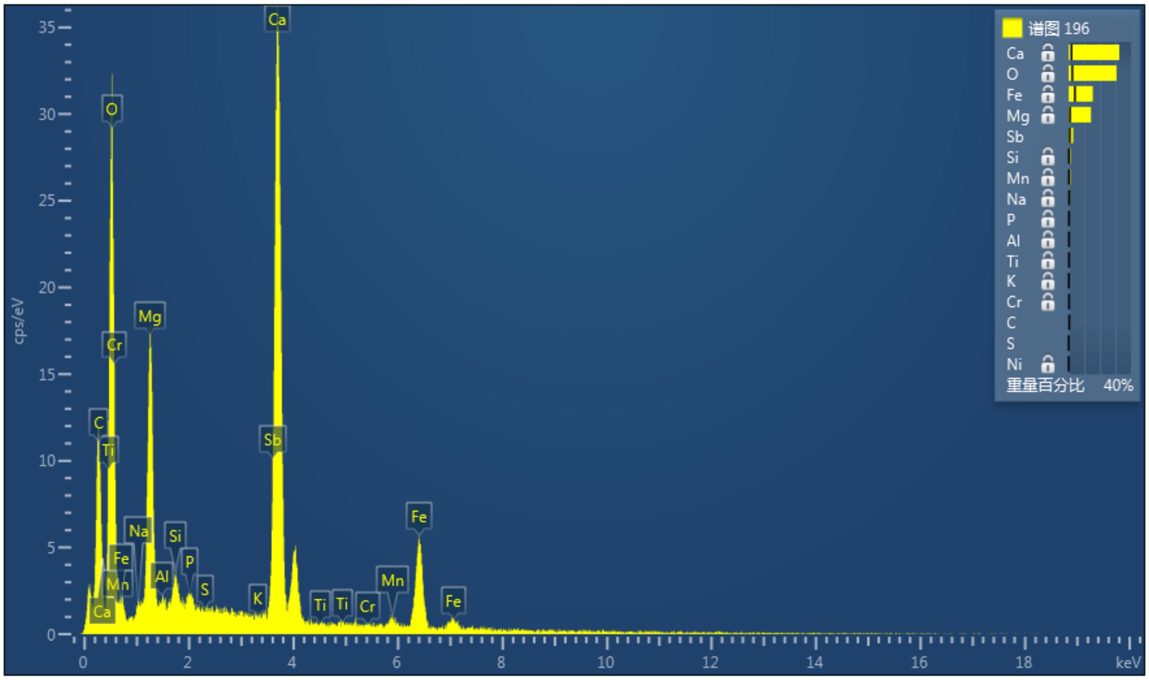

Supplementary Figure 7. EDS spectrum taken into the eggshell at Spot 2.

This spectrum shows an enrichment in Ca, Mg, O, and Fe.

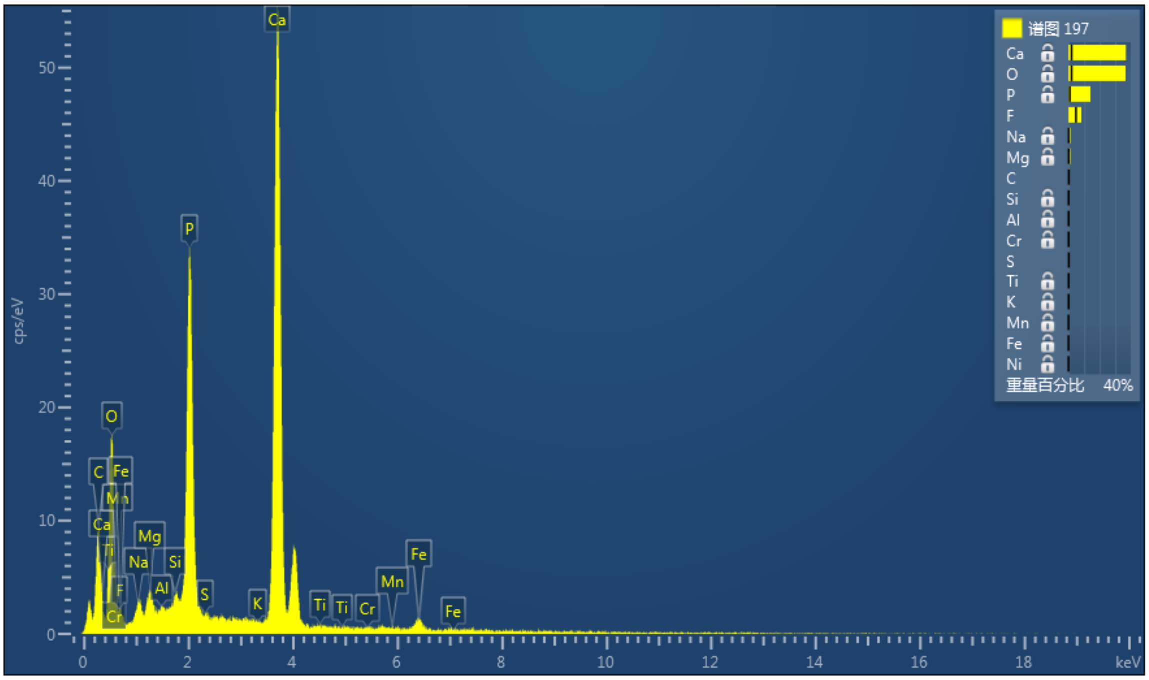

Supplementary Figure 8. EDS spectrum taken into the cuticle at Spot 3.

This spectrum shows an enrichment in P when compared to that of the eggshell spectra.

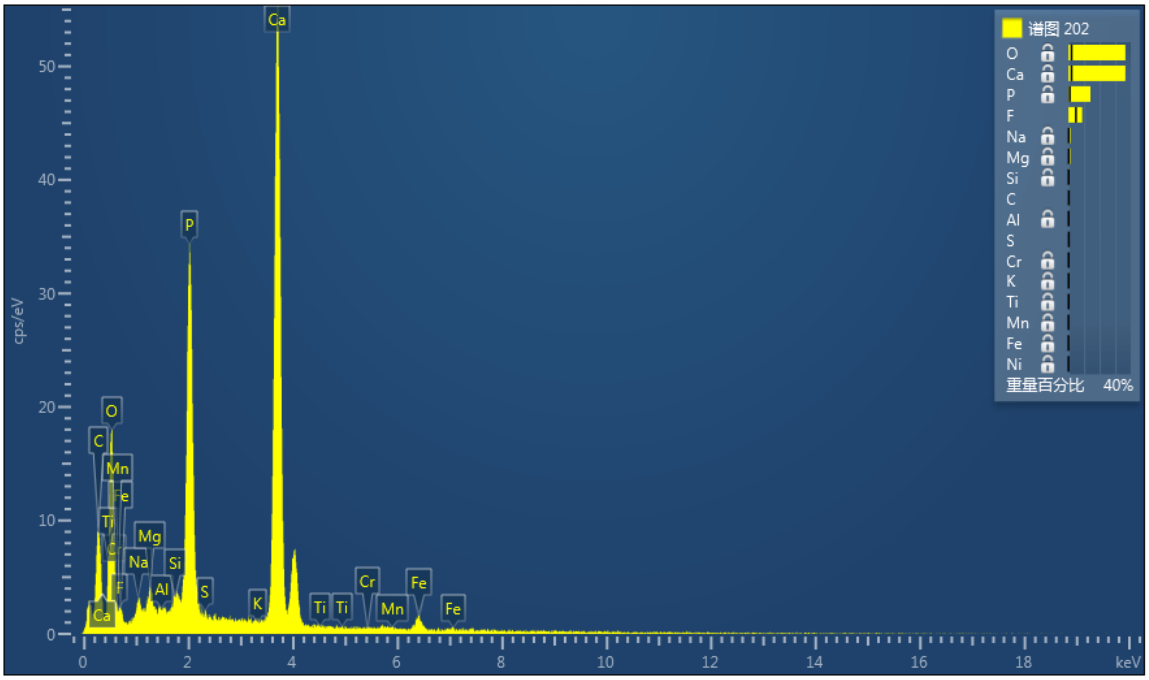

Supplementary Figure 9. EDS spectrum taken into the cuticle at Spot 4.

This spectrum shows an enrichment in P when compared to that of the eggshell spectra.

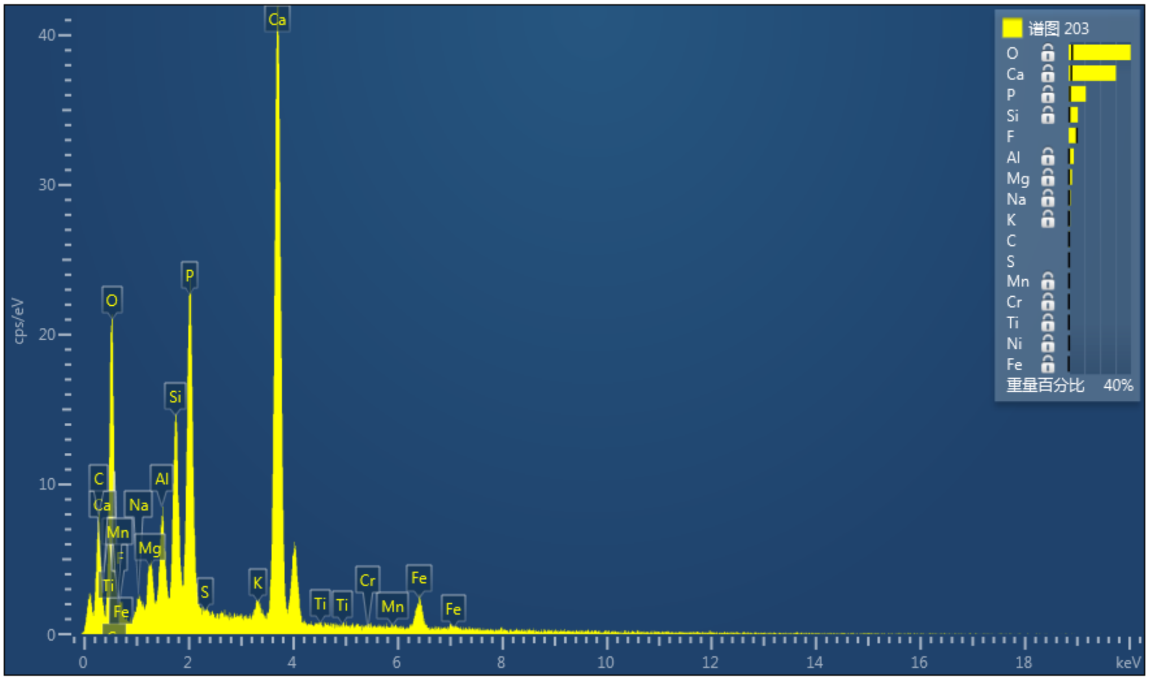

Supplementary Figure 10. EDS spectrum taken into the sediment at spot 5.

This spectrum shows a high proportion of Ca, Pa, O and Si. The proportion of Ca is high because the Changma locality is mostly composed of lacustrine carbonates[8].

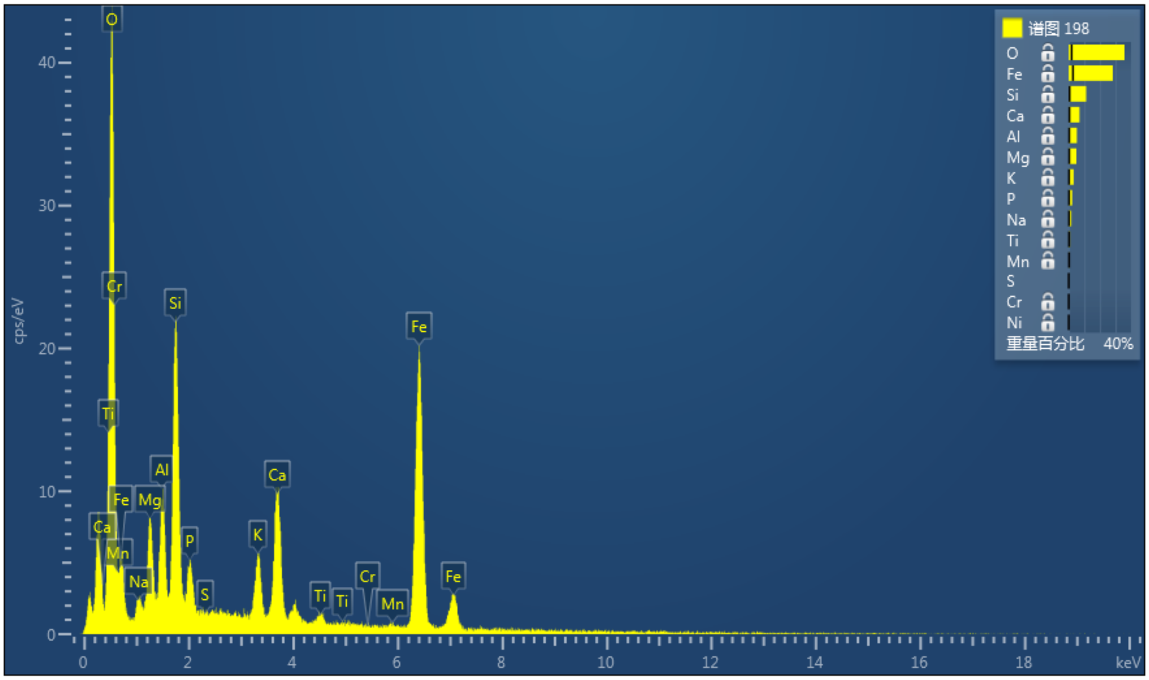

Supplementary Figure 11. EDS spectrum taken into the shell membrane at Spot 6. This spectrum is distinct in being enriched in Si, O and Fe. Because there is no notable peak of carbon very little of the original organic material is left.

Supplementary Table 1. Proportions of P, Ca, and Si compared between the cuticle, eggshell and sediment.

Areas	P(wt%)	P(wt % Sigma)	Ca(wt%)	Ca(wt % Sigma)	Si(wt %)	Si(Wt % Sigma)	**Ca/Si**
Cuticle (spot 3)	14.02	0.76	36.80	1.93	0.47	0.09	78.30
Eggshell (spot 2)	0.56	0.17	32.47	1.76	1.22	0.14	26.61
Sediment (spot 5)	10.96	0.64	30.24	1.67	5.84	0.35	5.18

Note: Wt% means percentage by weight and wt% sigma is the error bar of the wt%. No significant differences in the spectra of spots 3 and 4 (both from the cuticle) were observed, therefore in this table only one of them is shown (spot 3); and similarly, no significant differences in the spectra from spots 1 and 2 (both from the eggshell layers) were observed so only spot 2 is shown here.

Supplementary References

1 Atterholt, J., Hutchison, J. H. & O'Connor, J. K. The most complete enantiornithine from North America and a phylogenetic analysis of the Avisauridae. *PeerJ* **6**, e5910 (2018).

2 O'Connor, J. K., Averianov, A. O. & Zelenkov, N. V. A confuciusornithiform (Aves, Pygostylia)-like tarsometatarsus from the Early Cretaceous of Siberia and a discussion of the evolution of avian hind limb musculature. *J. Vertebr. Paleontol.* **34**, 647-656 (2014).

3 O'Connor, J. K. *A systematic review of Enantiornithes (Aves: Ornithothoraces)*, University of Southern California, (2009).

4 Chiappe, L. M. Enantiornithine (Aves) tarsometatarsi from the Cretaceous Lecho Formation of northwestern Argentina. Am. Mus. novit. 3083. (1993).

5 You, H.-L. *et al.* A second Cretaceous ornithuromorph bird from the Changma Basin, Gansu Province, northwestern China. *Acta Palaeontol. Pol.* **55**, 617-625 (2010).

6 Ji, S.-A. *et al.* A new, three-dimensionally preserved enantiornithine bird (Aves: Ornithothoraces) from Gansu Province, north-western China. *Zool. J. Linn. Soc.* **162**, 201-219 (2011).

7 Yang, T.-R., Chen, Y.-H., Wiemann, J., Spiering, B. & Sander, P. M. Fossil eggshell cuticle elucidates dinosaur nesting ecology. *PeerJ* **6**, e5144 (2018).

8 Suarez, M. B., Ludvigson, G. A., González, L. A., Al-Suwaidi, A. H. & You, H.-L. Stable isotope chemostratigraphy in lacustrine strata of the Xiagou Formation, Gansu Province, NW China. *Geol. Soc., London, Special Publications* **382**, SP382. 381 (2013).

9 D'Alba, L., Maia, R., Hauber, M. E. & Shawkey, M. D. The evolution of eggshell cuticle in relation to nesting ecology. *Proc. R. Soc. B* **283**, 20160687 (2016).

10 D'Alba, L. *et al.* What Does the Eggshell Cuticle Do? A Functional Comparison of Avian Eggshell Cuticles. *Physiol. Biochem. Zool.* **90**, 588-599 (2017).

11 Fernández, M. S. & Salgado, L. The youngest egg of avian affinities from the Cretaceous of Patagonia. *Hist. Biol.* 1-9 (2018).

12 Folk, R. L. & Leo Lynch, F. Organic matter, putative nannobacteria and the formation of ooids and hardgrounds. *Sedimentology* **48**, 215-229 (2001).

13 Schweitzer, M., Chiappe, L., Garrido, A., Lowenstein, J. & Pincus, S. Molecular preservation in Late Cretaceous sauropod dinosaur eggshells. *Proc. R. Soc. Lond.* B. *Biol. Sci*. **272**, 775-784 (2005).

14 Fernández-Jalvo, Y. *et al.* Early bone diagenesis in temperate environments: Part I: Surface features and histology. *Palaeogeogr. Palaeoclimatol. Palaeoecol.* **288**, 62-81 (2010).

15 Ponton, F. *et al.* Variation of the outer circumferential layer in the limb bones of birds. *Acta Ornithol.* **39**, 137-140 (2004).
